# Measuring treatment response in psoriatic arthritis: comparison between PSoriatic Arthritis Response Criteria and Disease Activity Score

**DOI:** 10.1093/rap/rkag120

**Published:** 2026-10-08

**Authors:** Khadijah Patel, Moshiur Rahman Khasru, Nisha Nair, Maarten De Wit, Arnon Katz, Charlotte Thompson, Meghna Jani, Hector Chinoy, Anne Barton, Philippa Curry, James Bluett, for the OUTPASS Collaborators

**Affiliations:** Division of Musculoskeletal and Dermatological Sciences, Faculty of Biology, Medicine and Health, University of Manchester, Manchester, UK; Division of Musculoskeletal and Dermatological Sciences, Faculty of Biology, Medicine and Health, University of Manchester, Manchester, UK; Division of Musculoskeletal and Dermatological Sciences, Faculty of Biology, Medicine and Health, University of Manchester, Manchester, UK; NIHR Manchester Biomedical Research Centre, Manchester University NHS Foundation Trust, Manchester Academic Health Sciences Centre, Manchester, UK; GRAPPA Patient Research Partner, The Netherlands; GRAPPA Patient Research Partner, Israel; Department of Rheumatology, University Hospitals Sussex NHS Foundation Trust, Sussex, UK; Division of Musculoskeletal and Dermatological Sciences, Faculty of Biology, Medicine and Health, University of Manchester, Manchester, UK; Division of Musculoskeletal and Dermatological Sciences, Faculty of Biology, Medicine and Health, University of Manchester, Manchester, UK; Department of Rheumatology, Salford Royal Hospital, Northern Care Alliance NHS Foundation Trust, Manchester Academic Health Science Centre, Salford, UK; Division of Musculoskeletal and Dermatological Sciences, Faculty of Biology, Medicine and Health, University of Manchester, Manchester, UK; Division of Musculoskeletal and Dermatological Sciences, Faculty of Biology, Medicine and Health, University of Manchester, Manchester, UK; Division of Musculoskeletal and Dermatological Sciences, Faculty of Biology, Medicine and Health, University of Manchester, Manchester, UK; NIHR Manchester Biomedical Research Centre, Manchester University NHS Foundation Trust, Manchester Academic Health Sciences Centre, Manchester, UK

**Keywords:** psoriatic arthritis, outcome measures, pain assessment, observational studies, biological therapies

## Abstract

**Objectives:**

Despite current UK guidelines recommending the use of the PSoriatic Arthritis Response Criteria (PsARC) to assess response to advanced targeted therapies in PsA patients, some clinicians use the 28-joint Disease Activity Score (DAS28), primarily developed for RA. Concordance of the two scores in PsA patients commencing advanced therapies has not previously been explored and is investigated here.

**Methods:**

Utilising data from the Outcomes of Treatment in Psoriatic Arthritis Study Syndicate (OUTPASS – RDN-CPMS 13910) cohort, a UK multicentre, observational study, components of PsARC and DAS28 scores were calculated at baseline (pretreatment), 3 months and 6 months following treatment, where available. DAS28-based good/moderate responses were combined and compared with PsARC responder status and agreement tested using the prevalence-adjusted bias-adjusted kappa test.

**Results:**

Of 413 patients, 78% were classified as responders by PsARC at 3 months and 73% at 6 months and 80% by DAS28 at both 3 and 6 months. Of 218 visits, where data were recorded for patients with both PsARC and DAS28 at 3 or 6 months, 17.9% were overall discordant. Prevalence-adjusted bias-adjusted kappa values revealed ‘substantial agreement’ (64.22%) between the two scores. Further analysis between the overall concordant/discordant populations showed significant differences with PsARC patient global assessments (PtGAs), (*P* = 0.02).

**Conclusion:**

PsARC and DAS28-based response criteria show ‘substantial agreement’, supporting the use of either score in patients commencing advanced therapies. PsARC PtGA was the sole significantly different (*P* < 0.05) subcomponent between the discordant and concordant groups, highlighting an importance in considering patient perspectives and psychosocial factors affecting response to treatment.

Key messages

- Lack of consensus in PsA treatment response could hinder effective clinical practice due to heterogeneity.
- PsARC and DAS28-based responses show ‘substantial’ agreement up to 6 months and could be used interchangeably.
- Significant differences in patient global assessments highlight a need to explore modifiable patient psychosocial factors.

## Introduction

PsA is a life-long chronic inflammatory-mediated disease. With a complex interplay of genetic, environmental and autoimmune contributors, the identification of a clear pathogenetic cause and effective therapeutic targets in PsA has remained a significant global challenge [1]. In the UK, the prevalence of PsA reportedly increased from 0.03% to 0.37% between 1991 and 2020, with a globally estimated 30% of psoriasis (PsO) patients later developing PsA [2–4]. Despite this, there is a growing understanding of disease pathogenesis leading to several advanced therapies available in PsA, including biologic and targeted synthetic DMARDs (bDMARDs and tsDMARDs, respectively) and small molecule inhibitors [1, 5]. To thoroughly utilise these advancements and ensure early implementation of effective interventions, it is necessary to establish globally standardised methods of measuring and monitoring disease activity.

This multifaceted disease is heavily characterised by a combination of dermatological and musculoskeletal manifestations. The majority of PsA patients have psoriasis, while arthritic peripheral joint inflammation presents as swelling, stiffness and persistent pain. The disease can include dactylitis, axial disease, nail dystrophy and/or enthesitis [6, 7]. The vast assortment of clinical manifestations and affected tissues present not only a significant diagnostic challenge but also a difficulty in accurately measuring PsA disease activity to inform treatment decisions.

Current EULAR guidelines recommend the use of validated measures of musculoskeletal disease activity in clinical practice to define the target and to guide treatment decisions [8, 9]. While disease activity scores have been developed for PsA, including the Psoriatic Arthritis Response Criteria (PsARC), Disease Activity in Psoriatic Arthritis (DAPSA), Psoriatic Arthritis Disease Activity Score (PASDAS) and measures of minimal disease activity or very low disease activity, non-PsA-specific scores such as the 28-joint DAS (DAS28) and US Food and Drug Administration–approved American College of Rheumatology (ACR) continue to be used in the clinic and clinical trials [10–12]. Additionally, the OMERACT international initiative has developed a core domain set for PsA involving musculoskeletal disease activity including peripheral joints, enthesitis, dactylitis and spine symptoms [13, 14]. The abundance of global tools for measuring treatment response produces a layer of disorganisation for researchers and clinicians by applying and integrating multiple assessments. The use of varying criteria and thresholds provides further difficulty when comparing treatment responses across a wider population, as well as a hindrance for developing consensus across international guidelines. A lack of consistency may also cause variations between institutions, potentially impacting both physicians’ and patients’ understanding of disease progression, how it should be managed and how treatment targets should best be monitored.

The DAS28, while developed for RA, is a frequently applied PsA-non-specific musculoskeletal response measurement comprising three or four components and assessing 28 key arthritic joints [15, 16]. These components comprise the clinically assessed swollen and tender joint counts (SJC, TJC), measured CRP or ESR and patient assessed general health on a 10-cm visual analogue scale, the Patient’s Global Assessment (PtGA) [17]. The DAS28-based response may be categorised as good, moderate or none, depending on the patient’s current and change in DAS28 score [18].

The DAS28 has wide use in multiple registries and clinical trials for PsA, including the Danish Nationwide Registry of Biological Therapies [19] and Norwegian Disease-Modifying Anti-Rheumatic Drug register [20], and studies by Nash *et al.* [21] and Linde *et al.* [22]. While widely used, it has not been approved as an outcome measure by the Group for Research and Assessment of Psoriasis and Psoriatic Arthritis and OMERACT due to its limited psychometric properties [23]. Limited data are available that directly compare DAS28-based outcome measures with other outcome measures developed for use in PsA. The PsARC is a PsA-specific response measurement used in the UK and recommended by the National Institute for Health and Care Excellence (NICE) to assess musculoskeletal drug response in patients commencing a bDMARD [24]. The criterion evaluates a broader 66 peripheral joint SJC and 68 joint TJC. Subcomponents measured include clinically assessed SJC, TJC and PtGA, and a Physician’s Global Assessment (PhGA), measured on a 0–5 Likert scale [25]. A positive response to treatment is classified by improvement in at least two subcomponents, including either the SJC or TJC, with no deterioration in any subcomponent [26].

This study aims to investigate similarities and differences between two commonly used response criteria, the PsARC and DAS28-based response in PsA patients commencing a targeted therapeutic and to evaluate differences in subcomponents when response criteria are discordant.

## Methods

### Study design

Patient data were sourced from the UK-based multicentre observational cohort Outcomes of Treatment in Psoriatic Arthritis Study Syndicate [OUTPASS (RDN-CPMS 13910)], as described elsewhere [27, 28]. Ethical approval for the study was granted by the National Research Ethics Service Committee Northwest–Greater Manchester Central (reference 13/NW/0068).

Participants were eligible to be recruited to OUTPASS if they met the following inclusion criteria: willing and able to give written consent, had PsA according to the CASPAR criteria or as diagnosed by a clinician [29], about to commence treatment with a biologic or small molecule inhibitor therapy, ≥18 years of age and had baseline (pretreatment) components of PsARC or DAS28 score available; this must be within 3 months of starting their biologic or small molecule inhibitor and up to 1 month before consent.

Patients with PsARC and DAS28 response criteria available at 3 or 6 months were included in the analysis. Patients either unwilling or unable to donate a blood sample or unable to provide informed consent or to participate in all follow-up visits were not included in this study. Missing data were not imputed and were excluded from the analysis (Supplementary Fig. S1).

### Clinical and demographic data collection

Patient and clinician questionnaires recorded age at study entry, age at diagnosis, sex, height, BMI, conventional synthetic DMARD therapy and tsDMARD therapy commencing at baseline. Additionally, 28/68 TJC, 28/66 SJC, PtGA (0–5 and 0–100), PhGA (0–5) and CRP/ESR were collected at each time point.

### Statistical analysis

All analyses were conducted using STATA version 14 (StataCorp, College Station, TX, USA) [30]. PsARC and DAS28 scores were calculated for each time point and used to classify patients as responders or non-responders to treatment.

DAS28 treatment response was calculated using the DAS28 response classification criteria, developed for RA, as previously described [11, 17, 31].

Good and moderate DAS28 responders were categorised as ‘responders’ and poor responders were categorised as ‘non-responders’ to facilitate comparability with the PsARC responder/non-responder classification, as carried out previously [27].

‘Concordant responders’ were classified as patients for whom both scores agree on the classification of responders to treatment. ‘Concordant non-responders’ were classified as patients for whom the two scores agree on the classification of non-responders to treatment. ‘Positive discordance’ was defined as patients categorised as responders by the DAS28 but not by PsARC. Conversely, ‘negative discordance’ was defined as patients categorised as responders by PsARC but not by the DAS28. ‘Overall concordance/discordance’ was used to refer to both two categories conjoined that agree (overall concordant) or disagree (overall discordant). Prevalence-adjusted bias-adjusted kappa (PABAK) was used to assess the level of agreement between the two DASs and the reliability in classifying treatment responders. Results were interpreted using a standardised scale (Supplementary Table S1) [32].

Comparison of the subcomponents, (SJC, TJC, PtGA, PhGA, CRP and ESR) of the PsARC and DAS28 composite scores between the two overall discordant and concordant groups at baseline was undertaken. Histograms were generated for all subcomponents within both groups to inform statistical testing. The Mann–Whitney U test was used for median-assessed skewed data and the unpaired *t*-test for mean-assessed normally distributed data.

The PsARC and DAS28 subcomponents were statistically analysed for significant difference among the overall discordant patients at baseline. The Wilcoxon signed-rank test was applied for non-normally distributed data and the paired *t*-test for normally distributed data.

Participants with oligoarticular disease, defined as ≤3 TJC and SJC at baseline, were also explored comparatively between the PsARC and DAS28 for effect on overall discordance at 3 months (*n* = 28) and 6 months (*n* = 19).

## Results

### Cohort characteristics

Patient baseline demographics are summarized in Table 1, with a mean BMI of 31.5 kg/m^2^ (s.d. 12.2), classifying the overall cohort as obese on average, with overall mild psoriasis [Psoriasis Area and Severity Index (PASI) 4.17 (s.d. 5.15)] and few [7.75% (*n* = 32)] with severe psoriasis (PASI ≥10) according to current NICE guidelines [33]. The mean age at diagnosis was 40 years and 48 years at study entry. The cohort was 55% female, with a mean baseline DAS28 score of 4.9 (s.d. 1.0).

**Table 1:** Patient clinical and demographic characteristics at baseline.

Characteristics	Overall cohort (% missing)	Overall concordant subgroup (% missing)	Overall discordant subgroup (% missing)
Patients, *n*	413 (0)	179 (0)	39 (0)
DAS28 at baseline, mean	4.9 (11.6)	4.9 (0.0)	4.7 0
DAS28 SJC:TJC, mean	5.5:9.6 (11.1:11.4)	5.3:9.3 (0.6:0.6)	4:9.1 (0:0)
PsARC SJC:TJC, mean	8.7:17.1 (4.8:6.5)	9.0:18.9 (0:0.6)	7.3:18.5 (0:0)
Age at start of study, years, mean	48 (1.0)	49 (1.1)	50 (0)
Female (%)	55.0 (1.0)	53.1 (1.1)	59.9 0
Height (cm)	71–199 (7.5)	88–193 (5.0)	155–183 (7.7)
Body weight (kg), mean	91.9 (4.6)	89.7 (3.9)	87.6 (7.7)
BMI (kg/m^2^), mean	31.5 (9.4)	31.4 (6.7)	30.5 (10.3)
PASI, mean [PASI≥10, n (%)]	4.17 (27.6) [*n* = 32 (7.75%)]	3.73 (25.14) [*n* = 11 (6.15%)]	5.08 (15.38) [*n* = 5 (12.8%)]

Details of patient therapies are provided in Table 2. All patients received advanced targeted therapies, of which 42.1% were prescribed adalimumab and 30.5% etanercept. Of all the patients, 87% were prescribed a concomitant DMARD, methotrexate being the most prescribed (49.7%).

**Table 2:** DMARDs and biologics taken at baseline.

DMARDs at baseline	*n* (**%**)	Biologics at baseline	*n* (%)
Methotrexate	179 (49.7)	Adalimumab	174 (42.1)
Sulfasalazine	113 (31.4)	Etanercept	126 (30.5)
Leflunomide	45 (12.5)	Secukinumab	33 (8.0)
Hydroxychloroquine	14 (3.9)	Ustekinumab	24 (5.8)
Ciclosporin	7 (1.9)	Golimumab	24 (5.8)
Azathioprine	1 (0.3)	Certolizumab	19 (4.6)
Myocrisin	1 (0.3)	Ixekizumab	5 (1.2)
Missing	53	Tofacitinib	3 (0.7)
Total	413 (100)	Other	3 (0.7)

### Comparison of PsARC and DAS28

There was a mean reduction in baseline DAS28 score of 1.79 by 3 months and 1.91 at 6 months. Based on the DAS-28 criteria, 180 of 225 patients (80%) were classified as responders at 3 months. This percentage remained the same at 6 months, with 142/178 patients (80%) classified as responders (Table 3).

**Table 3:** Frequency of DAS28/PsARC responders at 3 and 6 months.

Classification	3 months DAS28-based response	6 months DAS28-based response	3 months PsARC	6 months PsARC
Non-responder, *n* (%)	45 (10.90)	36 (8.72)	50 (12.11)	49 (11.86)
Responder, *n* (%)	180 (43.58)	142 (34.38)	179 (43.34)	132 (31.96)
Missing, *n* (%)	188 (45.52)	235 (56.90)	184 (44.55)	232 (56.17)
Total, *n* (%)	413 (100)	413 (100)	413 (100)	413 (100)

For PsARC, 179/229 patients (78%) were classified as responders at 3 months. At 6 months, 132/181 patients (73%) were classified as responders (Table 3).

Data were available for DAS28 and PsARC in 218 visits at 3 or 6 months. A collective comparison of PsARC and DAS28 response at 3 months revealed 72% of patients as concordant responders and 14% as concordant non-responders. Conversely, 14% of patients were overall discordant with 11 patients classified as negative discordance and 15 as positive discordance.

At 6 months, 68% were concordant responders, 17% concordant non-responders and 15% overall discordant, with 10 patients classified as negative discordance and 11 as positive discordance (Supplementary Tables S2 and S3).

### Oligoarticular analysis

A subanalysis of baseline oligoarticular disease demonstrated 67.86% concordant response and 7.14% concordant non-response when classifying response by the PsARC and DAS28 at 3 months (*n* = 28). Alternatively, they demonstrated 17.86% positive discordance and 7.14% negative discordance, resulting in an overall concordance of 75.0% and overall discordance of 25.0% (Supplementary Table S4).

At 6 months, 47.37% were classified as concordant response and 36.32% as concordant non-response, while 15.79% were classified as positive discordance and 10.53% as negative discordance, resulting in an overall concordance of 73.68% and overall discordance of 26.32% (Supplementary Table S4).

### PABAK statistical results

From a total sample of 218 patient visits, for which DAS28 and PsARC are both available in a single time point, at either 3 or 6 months of targeted therapy, 69.7% were classified as concordant responders, 12.4% as concordant non-responders and 17.9% as overall discordant (Table 4). Cohen’s kappa agreement between DAS28 and PsARC at either 3 or 6 months, where available, was observed at 82.11%, defined as 46.74% between random and perfect agreement (raw kappa). When further adjusting for PABAK, agreement between PsARC and DAS28 was found to be 64.22%, supporting ‘substantial agreement’ (SE 0.05). This was further supported by Gwet’s AC, resulting in 73.07% ‘substantial agreement’ when avoiding the kappa prevalence paradox (SE 0.04) (Table 5, Supplementary Table S1).

**Table 4:** Summary of responder/non-responder classification by PsARC and DAS28 at 3 or 6 months.

Classification	DAS28 non-responder, *n* (%)	DAS28 responder, *n* (%)	Total, *n* (%)
PsARC non-responder	27 (12.39)	22 (10.09)	49 (22.48)
PsARC responder	17 (7.80)	152 (69.72)	169 (77.52)
Total	44 (20.19)	174 (79.81)	218 (100)

Table summarising concordant responders (*n* = 152), concordant non-responders (*n* = 27), positive discordance (*n* = 22) and negative discordance (*n* = 17) using frequency (%) as classified by the PsARC and DAS28, as available for 3 or 6 months (*n* = 218).

**Table 5:** PABAK and Gwet’s AC.

	Coefficient (%)	SE	95% coefficient interval
Observed agreement	82.11	0.026	0.770–0.872
Cohen’s kappa	46.74	0.073	0.324–0.611
PABAK	64.22	0.052	0.540–0.744
Gwet’s AC	73.07	0.045	0.642–0.819

Table of results for PABAK supported by Gwet’s AC in assessing agreement between PsARC and DAS28 at either 3 or 6 months, where available. *n* = 218, PABAK = 64.22%.

### Response criteria subcomponent analysis

When comparing the overall concordant and overall discordant responders, a significant difference was observed for the PsARC PtGA [mean difference −0.40 (95% CI 0.06, 0.68), *P* = 0.02] and PsARC PhGA [mean difference −0.40 (95% CI 0.10, 0.72), *P* = 0.01] scores, with higher scores observed among the overall concordant groups (Table 6).

**Table 6:** Mann–Whitney U/unpaired *t*-test of subcomponents between overall discordant and overall concordant populations at baseline.

Subcomponents	Overall discordant		Overall concordant		Mann-Whitney U/unpaired *t*-test		
	Mean (s.d.)	Median (IQR) (s.d.)	Mean (s.d.)	Median (IQR) (s.d.)	Z statistic	T statistic (df)	*P*-value
DAS28 TJC	–	7.0 (3.0–16.0) (7.6)	–	8.0 (4 0.0–14.0) (6.3)	0.78	–	0.44
PsARC TJC	–	11.0 (6.0–26.0) (18.2)	–	15.0 (8.0–26.0) (15.1)	1.04	–	0.30
DAS28 SJC	–	4.0 (1.0–6.0) (3.4)	–	4.0 (3.0–7.0) (3.9)	1.86	–	0.06
PsARC SJC	–	5.0 (3.0–11.0) (6.4)	–	7.0 (4.0–12.0) (7.1)	1.77	–	0.08
DAS28 PtGA	62.2 (20.3)	–	65.5 (22.0)	–	–	0.86 (209)	0.39
PsARC PtGA	3.3 (0.8)	–	3.7 (0.9)	–	–	2.33 (215)	0.02
PsARC PhGA	3.2 (1.0)	–	3.6 (0.9)	–	–	2.58 (215)	0.01
CRP	–	10.0 (5.0–22.4) (18.8)	–	6.8 (4.0–17.0) (33.7)	−0.92	–	0.36
ESR	–	14.0 (7.0–34.0) (28.9)	–	17.0 (8 0.0–35.0) (24.9)	0.50	–	0.62

IQR: interquartile range; df: degrees of freedom.

## Discussion

In this UK multicentre observational study, we found 64.22% of cases in agreement between the DAS28-based and PsARC classification of responders. This ‘substantial’ agreement supports the use of either score when assessing musculoskeletal response for patients with PsA commencing an advanced targeted therapy in real-world clinical settings.

A previous study by Fransen *et al.* [26] reported better discrimination of drug from placebo when using the DAS28 compared with the PsARC in clinical trial data. However, the cohort size in that study was smaller (*n* = 164) and considered only 3–4 months. Further validation in larger independent datasets over a longer observation period should improve confidence in the findings.

Conversely, a study by Coates *et al.* [34] in 2013 explored concordance between differing joint counts used when measuring PsA. With a reported 21% of TJCs and 27% SJCs overlooked by the DAS28 in assessing PsA, the overall conclusion was drawn to endorse the use of 66/68 joint counts for a comprehensive and appropriate estimation of disease activity [34]. However, a sole focus on joint counts without consideration of other contributing composite measures, as used in routine clinical practice, may not be fully representative of its impact on the overall score and measure of overall disease activity. Other studies with similar conclusions include reviews by Mease [35] in 2008 and Ogdie *et al*. in 2021 [36]. However, concordance between the DAS28 and PsARC should be interpreted considering their different measurement properties. The DAS28 is a continuous multi-outcome domain instrument that captures the extent of change, whereas the PsARC is a composite outcome instrument that classifies patients as responders or non-responders using fixed thresholds [37, 38]. Therefore, patients meeting a PsARC response may have achieved different degrees of improvement, which is not reflected in a binary classification. Concordance between PsARC and DAS28 response does not necessarily indicate equivalent clinical improvement.

As PsA patients (≤50%) may experience a limited acute phase response, impact of CRP and ESR on DAS28 overall discordance was assessed and affirmed with no significant difference (*P* = 0.36 and *P* = 0.61, respectively). This aligns with the literature and the DAS28 formula, which assigns heavier weights to PtGA and TJC and less to CRP/ESR [39–41]. Here we found a significant difference in the PsARC PtGA (*P* = 0.02) and PhGA (*P* = 0.01) when assessing the subcomponents between the overall discordant and overall concordant groups. The discordance observed between the PtGA and PhGA may reflect differences in the aspects of disease captured by patient and physician assessments. The DAS28 and PsARC primarily measure joint or musculoskeletal disease activity and treatment response. They are not designed to comprehensively capture the full picture of disease activity nor the broader impact of PsA on patients. Factors not fully represented within these measures, including skin and nail disease, functional limitations and psychosocial factors such as occupational stress, sleep quality, mental health and social support, may therefore contribute to differences between patient and physician global assessments. However, these factors were not comprehensively captured in the present dataset and cannot be established as explanations for the observed discordance. Similarly, the severity of psoriasis on its own might not fully reflect its effect on patients, even though few patients had severe skin disease (defined as a PASI >10), as the involvement of visible or sensitive areas, such as the face or genital region, could still have a considerable impact on them. Since the patients’ perceived impact was not captured in detail, it was not possible to examine the contribution of such involvement to the differences between PtGA and PhGA. This conclusion is supported by Desthieux *et al.* [42] in a European multicentre cohort of 460 patients that assessed PsA patient-physician discordance in global assessment and found 85.8% of patients with PtGA > PhGA, reinforcing the need to further evaluate patient perspectives and the impact of ‘personal life’, ‘cultural factors’ and ‘psychological states’ that may be considered heavily by a patient when making an assessment of their disease burden but not by a physician.

In addition, a study published in 2019, by Kilic *et al.* [43], assessed patients with clinically defined remission and low disease activity for continued impact on quality of life. They found 22.2–49.3% patients still reporting ‘clinically important fatigue’ and 2.9–19.4% of patients experiencing ‘functional disability’ affecting quality of life despite the absence of inflammation or swollen joints. These findings highlight a potential need to broaden the scope of assessment beyond the current subcomponents, including patient psychosocial factors and potentially other neglected core domains in PsA, such as clinically relevant fatigue, that have been reported to contribute heavily to PsA disease burden and patient quality of life [23, 44, 45].

Further research is needed to compare the concordance of developed response criteria that include other disease activity measurements. For instance, the DAPSA, which is widely accepted, was validated by Schoels *et al.* in 2016 as a ‘feasible tool’ for PsA from an observational study with clinical trials and a response rate of 70%, but in a small cohort (*n* = 33). If international consensus is to be achieved, it would be worthwhile to assess and compare the treatment response against the PsA-specific score (PsARC) [46].

Alternatively, the PASDAS has been highlighted as ‘superior with respect to responsiveness’ in early PsA compared with the DAS 28 and DAPSA in a 2019 longitudinal Dutch study by Wervers *et al.* [47], where the PASDAS scored 1.00 effect size and a 0.95 standardised response mean while the DAPSA scored lowest, with 0.73 and 0.71, respectively [47]. However, although PASDAS components include OMERACT-approved core domains (joints, enthesitis, dactylitis and systemic biomarkers), the PASDAS also evaluates physical function using the SF-36 health survey, which can be challenging to implement due to time commitment and has not yet reached common clinical practice in the UK, where NICE guidelines currently endorse use of the PsARC and a moderate DAS28 response for PsA and do not necessarily reflect the depth of improvement achieved by an individual patient [13, 24, 47, 48]. This distinction is particularly important when comparing the PsARC, a binary response criteria, with the DAS28, a continuous measure in which the magnitude of change can be more readily quantified. Since the OMERACT core measures were published in 2016, 3 years after OUTPASS ethics approval, not all proposed variables have been collected and therefore the PASDAS cannot be assessed as part of this comparative study [14]. A further limitation is that despite being a clinically accepted standard in the UK, the PsARC and a moderate DAS28-based response, due to dichotomisation, may not fully represent treat to target/remission. Additionally, we were unable to explore the impact of the DAS28 not including the ankles/feet and upper limb disease in its assessment, as individual joint involvement was not collected in OUTPASS. Lastly, we were unable to reliably explore the effect of oligoarticular disease on discordance due to small sample sizes (3 months, *n* = 28; 6 months, *n* = 19) and missing data for some variables. Although the available data supported the planned analyses, missingness may have introduced bias, particularly if assessment availability differed systematically between patients. This may have influenced the observed relationships between patient- and physician-reported measures, as well as the estimated concordance between the PsARC and DAS28.

A major strength of this study is the use of real-world multicentre data from across the UK and assessment of two predominant DASs from a single time point, taken during the same appointment, reducing any potential bias.

## Conclusion

We have investigated the differences and overlap between two widely known and globally assessed musculoskeletal therapy response criteria (PsARC and DAS28) and have found considerable comparability in the scores. Despite this, significant discordance between the PtGA and PhGA highlights not only the subjectivity of the two composite scores but also the importance of understanding and accommodating skin and nail disease, patient lifestyle and mental health when assessing disease burden, and to strike a balance between a physician’s clinical examination and a patient’s impact on quality of life. However, these factors could not be directly evaluated within the present dataset and should therefore be considered hypotheses for future investigations rather than a demonstrated explanation. With very few studies available in this regard, it would be beneficial to further validate these findings and to assess the distribution and number of joints affected in the concordant and discordant populations. Other future projects may involve further comparative analyses and harmonisation of DASs developed for PsA, such as the DAPSA and PASDAS.

## Supplementary Material

rkag120_Supplementary_Data

## Data Availability

The data that support the findings of this study are available upon request from the corresponding author. The data are not publicly available due to privacy/ethical restrictions.
