# Supplementary material for: Measuring treatment response in psoriatic arthritis: comparison between PSoriatic Arthritis Response Criteria and Disease Activity Score
Source: Rheumatol Adv Pract. 2026 Oct 8;10(4):rkag120. doi: 10.1093/rap/rkag120 (PMC13759217; doi:10.1093/rap/rkag120)
Supplement: Supplementary file 1 [file rkag120_supplementary_data.docx]

**Supplementary Material:**

**Supplementary Table S1:** ***Cohen’s Kappa Test for Agreement Scale.***

*A standardised scale used to define and categorise the range of results outputted from a Cohen’s Kappa Test for Agreement from ≥ 0.0 poor to 0.81-1.00 almost perfect. Adapted from. (32).*

| **DAS-28**  **3M** | **PsARC 3M** | | | |
| --- | --- | --- | --- | --- |
|  | **Non-responder**  **(%)** | **Responder**  **(%)** | **Missing**  **(%)** | **Total**  **(%)** |
| **Non-responder (%)** | 25  (6.05) | 11  (2.66) | 9  (2.18) | 45  (10.90) |
| **Responder**  **(%)** | 15  (3.63) | 133  (32.20) | 32  (7.75) | 180  (43.58) |
| **Missing**  **(%)** | 10  (2.42) | 35  (8.47) | 143  (34.62) | 188  (45.52) |
| **Total**  **(%)** | 50  (12.11) | 179  (43.34) | 184  (44.55) | 413  (100) |

**Supplementary Table S2:** ***Comparison of responder/non-responder classification by PsARC and DAS-28, at 3M follow up.***

*A joint comparison of patients assessed by DAS-28 and PsARC at 3M follow up, with 80% classified as responders under DAS-28 and 78% as responders under PsARC. Of which, 72% are concordant responders and 14% are concordant non-responders, and 14% are overall discordant.*

*Imputation of response scores was not conducted.*

*-* *PsARC: Psoriatic Arthritis Response Criteria;* DAS-28:Disease Activity Score 28.

| **DAS-28**  **6M** | **PsARC 6M** | | | |
| --- | --- | --- | --- | --- |
|  | **Non-responder**  **(%)** | **Responder**  **(%)** | **Missing**  **(%)** | **Total**  **(%)** |
| **Non-responder (%)** | 23  (5.57) | 10  (2.42) | 3  (0.73) | 36  (8.72) |
| **Responder**  **(%)** | 11  (2.66) | 95  (23.00) | 36  (8.72) | 142  (34.38) |
| **Missing**  **(%)** | 15  (3.63) | 27  (6.54) | 193  (46.73) | 235  (56.90) |
| **Total**  **(%)** | 49  (11.86) | 132  (31.96) | 232  (56.17) | 413  (100) |

**Supplementary Table S3: *Comparison of responder/non-responder classification by PsARC and DAS-28 at 6M follow up.***

*A joint comparison of patients assessed by DAS-28 and PsARC at 6M follow up, with 78% classified as responders under DAS-28 and 76% as responders under PsARC. Of which, 68% are concordant responders and 17% are concordant non-responders, and 15% are overall discordant.*

*Imputation of response scores was not conducted.*

*-- DAS-28:Disease Activity Score 28; PsARC: Psoriatic Arthritis Response Criteria.*

| **OLIGOARTICULAR**  **DISEASE**  **3M** | **DAS-28**  **NON-RESPONDER (%)** | **DAS-28 RESPONDER (%)** | **Total**  **(%total)** |
| --- | --- | --- | --- |
| **PsARC NON-RESPONDER (%)** | 2 (7.14) | 5  (17.86) | 7  (25.00) |
| **PsARC RESPONDER (%)** | 2  (7.14) | 19 (67.86) | 21  (75) |
| **Total**  **(%total)** | 4  (14.28) | 14  (85.72) | 28  (100) |

| **OLIGOARTICULAR**  **DISEASE**  **6M** | **DAS-28**  **NON-RESPONDER (%)** | **DAS-28 RESPONDER (%)** | **Total**  **(%total)** |
| --- | --- | --- | --- |
| **PsARC NON-RESPONDER (%)** | 5 (26.32) | 3  (15.79) | 8  (42.11) |
| **PsARC RESPONDER (%)** | 2  (10.53) | 9  (47.37) | 11  (57.90) |
| **Total**  **(%total)** | 7  (36.85) | 12  (63.16) | 19  (100) |

**Supplementary Table S4:** ***Summary of responder/non-responder classification by PsARC and DAS-28 at 3M (left) and 6M (right), with oligoarticular disease.***

*(Left) The table describes the frequency/percentage of responders classified by DAS-28 and PsARC, for patients with oligoarticular disease (n=28). Overall discordance 25.0%. (Right) The table describes the frequency/percentage of responders classified by DAS-28 and PsARC for patients with oligoarticular disease (n=19). Overall discordance 26.3%.*

*-- DAS-28:Disease Activity Score 28; PsARC: Psoriatic Arthritis Response Criteria.*

**Supplementary Figure S1: Flow of participants and missing data throughout the study.**

*The figure displays the number of participants assessed at each stage, amount of missing data, and the flow of key research. Key values include 413 participants at start entry and 218 qualifying for comparative research.*

*- DAS-28:Disease Activity Score 28; PsARC: Psoriatic Arthritis Response Criteria; OUTPASS: Outcomes of Treatment in Psoriatic Arthritis Study Syndicate.*

**OUTPASS Collaborator List**

**Chief investigator: Dr James Bluett**

Wrightington, Wigan and Leigh Hospitals NHS Foundation Trust
Dr E G Chelliah (PI), Dr C Chattopadhyay (former PI)

Manchester Royal Infirmary (Manchester University NHS Foundation Trust)
Dr P Ho (PI), Prof. A Barton (former PI), Dr M Castelino, Prof. I Bruce, Dr R Gorodkin, Dr K Hyrich, Dr B Parker

Salford Royal (Northern Care Alliance NHS Foundation Trust)

Dr H Chinoy (PI), Prof. T O’Neil, Prof. A Herrick, Prof. A Jones, Dr R Cooper, Prof. W Dixon, Dr B Harrison, Dr Meghna Jani, Dr Audrey Low

Royal United Hospitals Bath NHS Foundation Trust

Dr E Korendowych (PI), Prof. N McHugh, Dr W Tillett

Aintree University Hospital (Liverpool University Hospitals NHS Foundation Trust)

Dr N Goodson (PI)

East Suffolk and North Essex NHS Foundation Trust

Dr S Lane (PI), Dr L Shand

Nottingham University Hospitals NHS Trust

Dr I Pande (PI), Dr I Gaywood (former PI), Dr F Rees, Dr M Rutter, Dr S Hayat, Dr J F McHale, Dr A C Jones, Dr P Lanyon, Dr A Gupta, Dr P A Courtney, Dr A Srikanth, Dr A Abhishek

Royal Devon University Healthcare NHS Foundation Trust

Dr S Kyle (PI), Dr R Manhas

Mid and South Essex NHS Foundation Trust

Dr A Nandagudi (PI), Dr S Selvan (former PI), Dr A Bharadwaj, Dr N Gendi, Dr R Alshakh

Fairfield Hospital (Northern Care Alliance NHS Foundation Trust)

Dr S Naz (PI), Dr M Ahmad (former PI)

North Manchester General Hospital (Manchester University NHS Foundation Trust)

Dr L Das, Dr M Pattrick, Dr A P Bowden, Dr E E Smith, Dr P Klimiuk, Dr D J Speden

University Hospitals of Morecambe Bay NHS Foundation Trust

Dr M Bukhari (PI), Dr S Kavaklieva, Dr L Ottewell, Dr M Massarotti

Midlands Partnership NHS Foundation Trust

Dr J Packham (PI)

Wythenshawe Hospital (Manchester University NHS Foundation Trust)

Dr P Watson (PI), Dr P Sanders (former PI), Dr S Haque, Dr B Pal, Dr E Bruce

The Mid Yorkshire Hospitals NHS Trust

Dr Z Karim (PI)

Torbay and South Devon NHS Foundation Trust

Dr K Mackay (PI), Dr H Shiels

Northampton General Hospital NHS Trust

Dr J Taylor (PI)

Stockport NHS Foundation Trust

Dr C Filer (PI), Dr A Ismail, Dr L Mercer

North Cumbria Integrated Care NHS Foundation Trust

Dr A Hassan (PI), Dr A Russell

University Hospitals of Leicester NHS Trust

Dr M Durrani (PI), Dr W Hassan (former PI), Dr A Samanta, Dr P Sheldon, Dr J Francis, Dr A Kinder, Dr R Neame, Dr A Moorthy

Barts Health NHS Trust

Prof. M Bombardieri (PI), Dr S Kelly (former PI)

Sheffield Teaching Hospitals NHS Foundation Trust

Dr J Maxwell (PI), Dr M Akil, Dr S Till, Dr L Dunkley, Dr R Tattersall, Dr R Kilding, Dr T Tait, Dr K-P Kuet, Dr B Grant, Dr M Kazmi

St Helens and Knowsley Teaching Hospitals NHS Trust

D Graham (PI), Dr V E Abernethy, Dr A R Clewes, Dr J K Dawson

NHS Greater Glasgow and Clyde

Dr S Siebert (PI), Dr G Fragoulis

Royal Liverpool and Broadgreen (Liverpool University Hospitals NHS Foundation Trust)

Dr D Mewar (PI), Dr E J Tunn, Dr K Nelson, Dr T D Kennedy, Dr C Dubois

The Dudley Group NHS Foundation Trust

Dr K Douglas (PI), Dr E Ladoyanni, Dr C Koutsianas, Dr N Erb, Dr R Klocke, Dr A J Whallett, Dr A Pace, Dr R Sandhu, Dr H John

Portsmouth Hospitals University NHS Trust

Dr S A Young Min (PI), Dr A Cooper, Dr J M Ledingham, Dr R G Hull, Dr F McCrae, Dr Wong, Dr Shaban

Mid Cheshire Hospitals NHS Foundation Trust

Dr K Putchakayala (PI)

The Rotherham NHS Foundation Trust

Dr R Kumari (PI), Dr G Smith (former PI)

South Warwickshire NHS Foundation Trust

Dr C Marguerie (PI)

Homerton Healthcare NHS Foundation Trust

Dr P Reynolds (PI), Dr C Thornton (former PI), Dr C Gorman, Dr C Murphy

Tameside and Glossop Integrated Care NHS Foundation Trust

Dr D Roy (PI)

Lancashire and South Cumbria NHS Foundation Trust

Dr S Horton (PI)

University College London Hospitals NHS Foundation Trust

Dr M Castelino (PI)

Sherwood Forest Hospitals NHS Foundation Trust

Dr David Walsh (PI)

Barnsley Hospital NHS Foundation Trust

Dr Victoria Bejarano (PI)

University Hospitals Sussex NHS Foundation Trust

Dr Charlotte Thompson (PI)

Airedale NHS Foundation Trust

Kelly Tempest (PI)

Pennine MSK Partnership

Dr James Bluett (PI)

University Hospitals Plymouth NHS Trust

Dr Ognjenka Savanovic-Abel (PI)
